# Validation of prognostic scores to predict short-term mortality in patients with HBV-related acute-on-chronic liver failure: The CLIF-C OF is superior to MELD, CLIF SOFA, and CLIF-C ACLF

**DOI:** 10.1097/MD.0000000000006802

**Published:** 2017-04-28

**Authors:** Ning Li, Chong Huang, Kang-Kang Yu, Qing Lu, Guang-Feng Shi, Jian-Ming Zheng

**Affiliations:** Department of Infectious Diseases, Huashan Hospital, Fudan University, Shanghai, China.

**Keywords:** ACLF grade, acute-on-chronic liver failure, CLIF consortium ACLF score, CLIF consortium organ failure score, CLIF sequential organ failure assessment score, hepatitis B virus, model for end-stage liver disease score

## Abstract

Acute-on-chronic liver failure (ACLF) in chronic hepatitis B (CHB) patients has a high short-term mortality. Identification of effective models to predict the short-term mortality may enable early intervention and improve patients’ prognosis. We aim to assess the performance of the CLIF Consortium Organ Failure score (CLIF-C OFs), CLIF sequential organ failure assessment score (CLIF-SOFAs), CLIF Consortium ACLF score (CLIF-C ACLFs), ACLF grade, and model for end-stage liver disease score (MELDs) in predicting the short-term mortality in CHB patients with ACLF.

Among the 155 consecutive adult patients with liver failure as a discharge diagnosis were screened, and all the patients were treated at the Department of Infectious Diseases, Huashan Hospital, Fudan University (Shanghai, China) from January 2010 to February 2016. The diagnosis of ACLF was based on the criteria formalized by the ACLF consensus recommendations of the Asian Pacific Association for the Study of the Liver (APASL). Diagnostic accuracy for predicting short-term (28-day) mortality was calculated for CLIF-C OFs, CLIF-SOFAs, CLIF-C ACLFs, ACLF grade, and MELDs in all patients.

One hundred fifty-five consecutive adult liver failure patients were screened and 85 patients including 73 males and 12 females were enrolled. Overall, the 28-day transplant-free mortality was 32% in all patients, and 100% in those with severe early course (ACLF-3). The area under the receiver operating characteristic curve (AUROC) of CLIF-C OFs (AUROC: 0.906, *P* = .0306, compared with MELDs) was higher than those of CLIF-SOFAs (AUROC: 0.876), CLIF-C ACLFs (AUROC: 0.858), ACLF grade (AUROC: 0.857), and MELDs (AUROC: 0.838) for predicting short-term mortality. The cut-point for baseline CLIF-C OFs in predicting death was 8.5, with 67% sensitivity, 90% specificity, and AUROC of 0.906 (95% CI: 0.8450–0.9679).

The results indicate that short-term mortality is high in patients with ACLF and CLIF Consortium Organ Failure score is superior to MELD, CLIF SOFA, and CLIF-C ACLF in predicting its short-term mortality.

## 1 Introduction

An estimated 240 million persons are chronically infected with hepatitis B virus (HBV) worldwide.^[1]^ HBV is one of the major cause of chronic liver disease. Accurately predicting the prognosis of chronic hepatitis B (CHB) patients with end-stage liver disease is crucial in therapeutic decision-making, especially when prioritizing organ allocation for liver transplantation (LT).

Acute-on-chronic liver failure (ACLF) is an increasingly recognized entity characterized by an acute deterioration of known or unknown chronic liver disease, or an acute decompensation of an end-stage liver disease, frequent requirement of organ supports, and high short-term mortality.^[2–7]^ Early recognition of such patients is mandatory, so that appropriate management is not delayed.^[8]^ The model for end-stage liver disease (MELD) is a conventional scoring system as prognostic tool devised for end-stage liver disease and the utility of transplantation.^[9]^ However, the ACLF definition and diagnostic criteria were recently proposed by the European Association for the Study of the Liver-Chronic Liver Failure (EASL-CLIF) Consortium. The CANONIC study has assessed the currently available prognostic scoring systems and developed a novel scoring system for the prognosis of patients with ACLF and acute decompensation.^[10,11]^ The ACLF patients were elevated by the CLIF Consortium ACLF score (CLIF-C ACLFs), incorporating the CLIF-C organ failure score (CLIF-C OFs), MELD, Child–Turcotte–Pugh (CTP), and so on. Ultimately, the CLIF-C ACLFs has since been independently validated with proven superior prognostic accuracy for ACLF compared with conventional scoring systems.^[10]^ However, the major etiology of cirrhosis was alcohol or HCV in that study. Different causes may be associated with different outcomes. Here, we mainly focus on ACLF caused by HBV infection rather than ACLF caused by alcohol or HCV; this will increase our knowledge about the utility of an established scoring system in a specific disease. Thus, we aimed to determine the performance of various prognostic scores including the CLIF-C OFs, CLIF sequential organ failure assessment score (CLIF-SOFAs), CLIF-C ACLFs, ACLF grade, and MELD in predicting the short-term (28-day) mortality in CHB patients with ACLF in this study.

## 2 Methods

### 2.1 Patients

**Figure 1:**
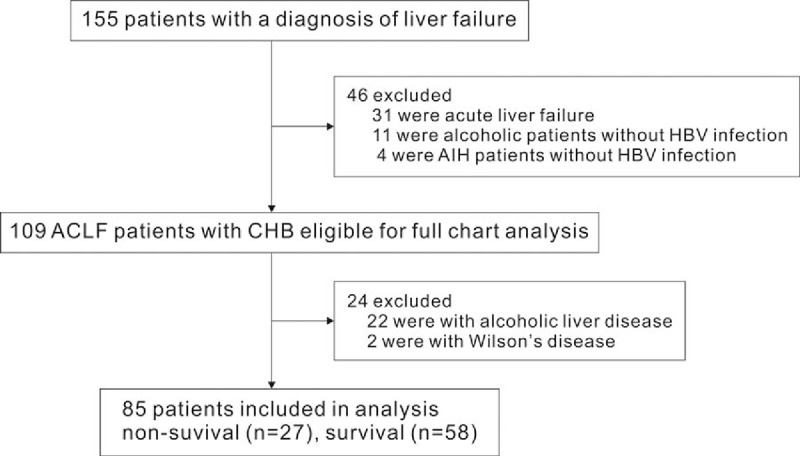
Flowchart for patients’ selection. HBV = hepatitis B virus.

Among the 155 consecutive adult patients with liver failure as a discharge diagnosis were screened, who were treated at the Department of Infectious Diseases, Huashan Hospital, Fudan University (Shanghai, China) from January 2010 to February 2016. Exclusion criteria were: patients aged less than 14 years; patients coinfected with human immunodeficiency virus; those with the coexistence of liver injury caused by any other etiologies including hepatitis C or D virus infection, drug intake, alcohol consumption, and autoimmune hepatitis, and so on; pregnancy and lactation. In all, 85 patients including 73 males and 12 females were finally enrolled into the study group (Fig. 1). The diagnosis of ACLF was based on the criteria formalized by the ACLF consensus recommendations of the Asian Pacific Association for the Study of the Liver (APASL) 2014.^[3]^ Acute liver failure is generally defined as development of hepatic encephalopathy within 4 weeks of onset of jaundice.^[3]^ Since the basic premise in ACLF is to identify patients with chronic liver disease or cirrhosis presenting as acute liver failure, the time frame for liver failure was kept as 4 weeks. Acute on chronic liver failure is defined as coagulation abnormality usually with an INR≥1.5 and bilirubin≥10 mg/dL in this study. The study was performed in accordance with the Helsinki Declaration and was approved by the Ethical Committee of Huashan Hospital, Fudan University.

### 2.2 Clinical characteristics and biochemical parameters

Clinical characteristics including jaundice, ascites, encephalopathy, or their combination were recorded. Components of various prognostic scores, such as body temperature, respiratory rate, neurological status, cardiac status (heart rate, blood pressure, mean arterial pressure), and blood parameters (routine blood tests with white blood cells and platelet counts, hematocrit, coagulation profiles including prothrombin time and INR, serum electrolyte levels, liver and renal function tests, and arterial blood gas analysis), were analyzed. Clinical characteristics including all the components of various prognostic scores and blood parameters (routine blood tests, coagulation function tests, serum electrolyte levels, liver and renal function tests, and arterial blood gas analysis) were analyzed.

### 2.3 Prognostic scores

All patients were evaluated for various prognostic scores including the CLIF-C OFs, CLIF-SOFAs, CLIF-C ACLFs, ACLF grade, and MELDs.^[9–11]^ Diagnostic criteria of ACLF grades were those previously described.^[11]^ The CLIF-C OFs (range 6–18) and CLIF-SOFAs (range 0–24) are proposed to evaluate organ failures in ACLF patients. The CLIF-C OFs at diagnosis was defined by the presence of hepatic, renal, cerebral, coagulatory, circulatory, and respiratory failure. Liver failure was classified by bilirubin<6 mg/dL, bilirubin≥6 mg/dL and <12 mg/dL, and bilirubin≥6 mg/dL, as subscore = 1, 2, and 3, respectively. Renal failure was classified by creatinine <2 mg/dL, creatinine ≥2 mg/dL and <3.5 mg/dL, and creatinine ≥3.5 mg/dL or renal replacement, as subscore = 1, 2, and 3, respectively. Cerebral failure was classified by grade 0, grade 1 and 2, and grade 3 and 4 (West–Haven grade for hepatic encephalopathy), as subscore = 1, 2, and 3, respectively. Coagulatory failure was classified by INR <2.0, INR≥2.0 and <2.5, and INR≥2.5, as subscore = 1, 2, and 3, respectively. Circulatory failure was classified by mean arterial pressure ≥70 mm Hg, mean arterial pressure <70 mm Hg, and use of vasopressors, as subscore = 1, 2, and 3, respectively. Respiratory failure was classified by PaO2/FiO2 > 300 or SpO2/FiO2 > 357, PaO2/FiO2≤300, and > 200 or SpO2/FiO2 > 214 and ≤357, and PaO2/FiO2≤200 or SpO2/FiO2≤214, as subscore = 1, 2, and 3, respectively. The CLIF-C ACLFs was calculated as: 10 × [0.33 × CLIF-C OFs + 0.04 × Age + 0.63 × ln(WBC count) − 2]. ACLF grade at diagnosis was defined by the presence of kidney failure (serum creatinine≥2 mg/dL) or other organ/system failures (hepatic, cerebral, coagulatory, circulatory, and respiratory). ACLF grade1 (ACLF-1) was the presence of kidney failure or other single organ/system failures, ACLF grade 2 (ACLF-2) and ACLF grade 3 (ACLF-3) were defined by the presence of 2 or ≥3 organ failures, respectively.^[12]^ MELD score was calculated as follows: 9.6 × ln[creatinine (mg/dL)] + 3.8 × ln[bilirubin (mg/dL)] + 11.2 × ln(INR) + 6.4 × (etiology: 0 if cholestatic or alcoholic, 1 otherwise).^[9]^

### 2.4 Statistical analysis

Statistical analyses were performed with the Graphpad 5.0 (Graphpad Software, San Diego, CA) and STATA 8.0 (College Station, TX). Variables were expressed as mean ± standard deviation unless otherwise specified. Survival probabilities were estimated by means of Kaplan–Meier method and were compared by the log-rank test. The performance of prognostic scores on the prediction of short-term mortality was assessed by the receiver operating characteristic curve. Differences in the parameters were compared using the nonparametric Mann–Whitney *U* test. A 2-tailed *P* value of <.05 was considered statistically significant.

## 3 Results

All patients were Asian. Table 1 shows the baseline characteristics at enrollment of the whole group during the first day after hospital admission. At enrollment, there were 23 patients (27%) in no ACLF group, 40 patients (47%) in ACLF grade 1 group, 13 patients (15%) in ACLF grade 2 group, and 9 patients (11%) in ACLF grade 3 group. In our cohort, all the patients received general supportive treatment. Thirty-four patients received lamivudine monotherapy, 21 patients received combination therapy with lamivudine and adefovir, 17 patients were treated with entecavir, 4 patients were treated with telbuvidine, 2 patients were treated with entecavir combined with adefovir, 2 patients were treated with tenofovir, 1 patient was treated with adefovir, and 4 patients received no antiviral treatment. Three patients performed liver transplantation finally.

**Table 1:**
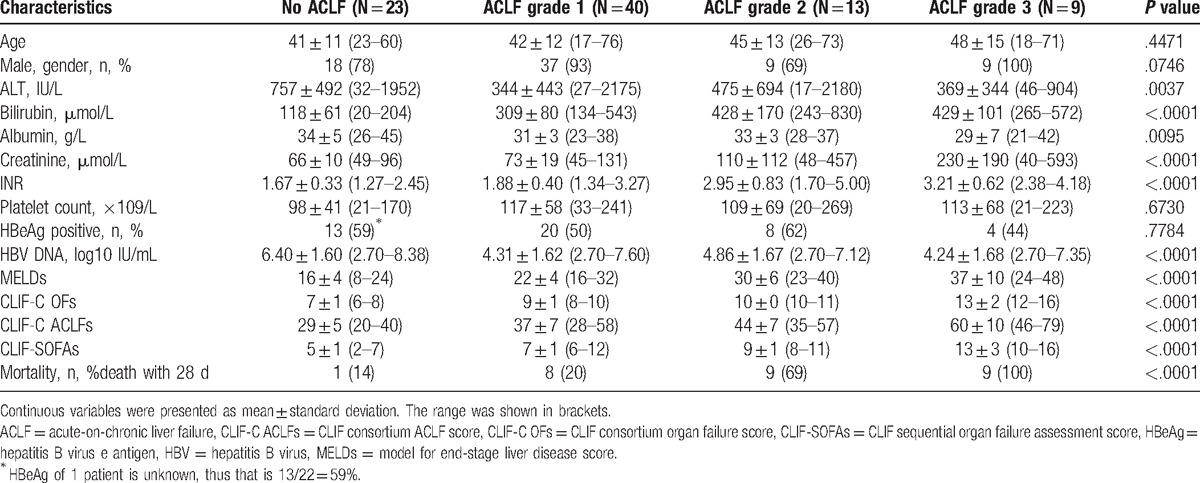
Baseline characteristics of the enrolled patients with ACLF.

Prognostic scores differed significantly in the no ACLF group and for ACLF grades 1 to 3 group respectively (CLIF-C OFs: 7, 9, 10, and 13; CLIF-C ACLF: 29, 37, 44, and 60; CLIF-SOFAs: 5, 7, 9, and 13; MELDs: 16, 22, 30, and 37).

The findings for gender, baseline ALT, platelets, HBeAg status, and HBV DNA level were similar for the survivors and nonsurvivors groups. Patients in the nonsurvivors group were older than those in the survivors group, had a higher level of bilirubin, creatinine and INR, and had a lower level of albumin (Table 2). Other than older age, lower albumin, lower serum sodium, higher bilirubin, higher creatinine, and higher INR were associated with short-term mortality. By the Cox-proportional logistic regression model, high INR and lower albumin remained as independent factors associated with overall mortality (Table 3).

**Table 2:**
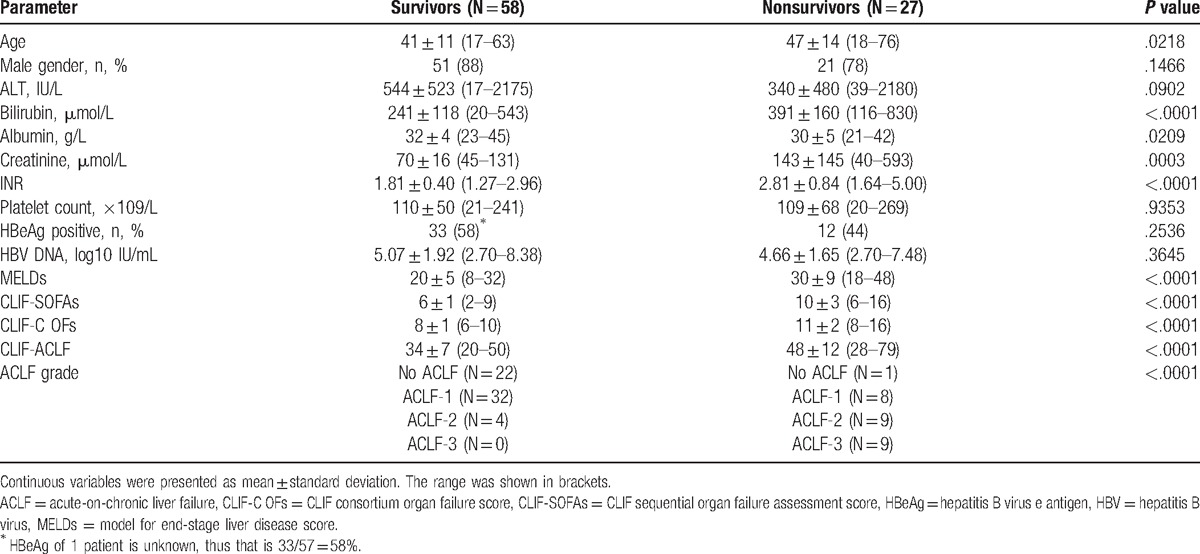
Comparison of survivors and nonsurvivors with ACLF.

**Table 3:**
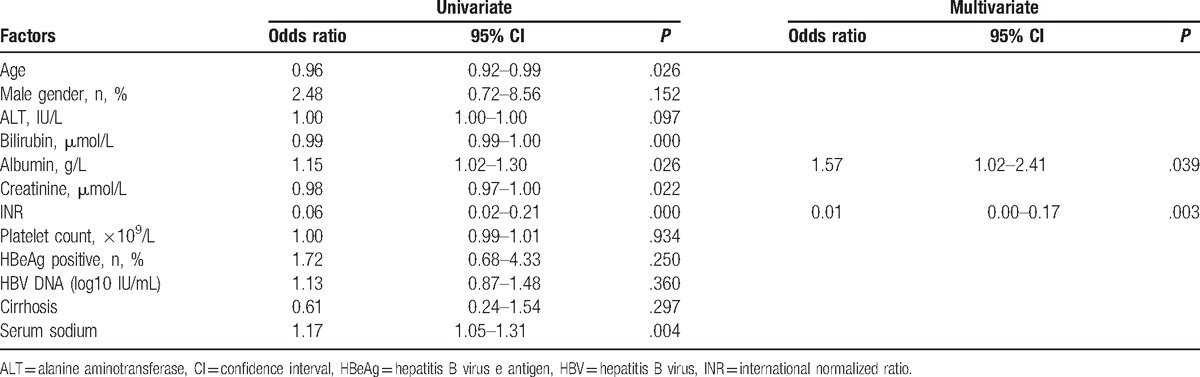
Factors associated with transplant-free mortality within 28 days.

**Figure 2:**
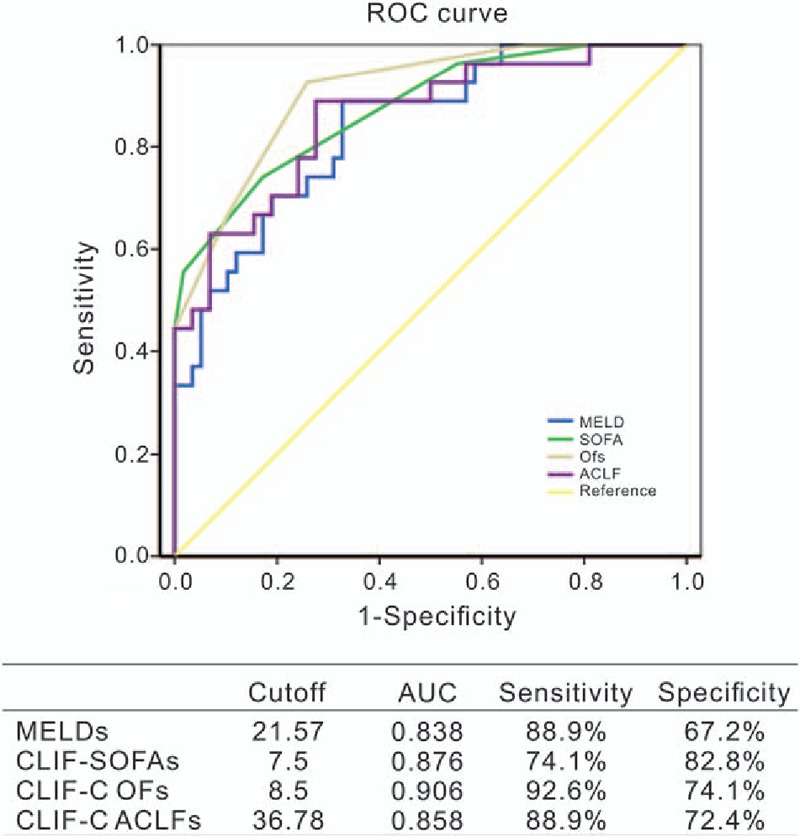
Accuracy of the CLIF-OFs as compared with that of MELD, CLIF SOFAs, and CLIF-ACLF in predicting 28-day mortality of the CHB patients with ACLF. ACLF = acute-on-chronic liver failure, CLIF SOFAs = CLIF sequential organ failure assessment score, MELDs = model for end-stage liver disease score.

**Figure 3:**
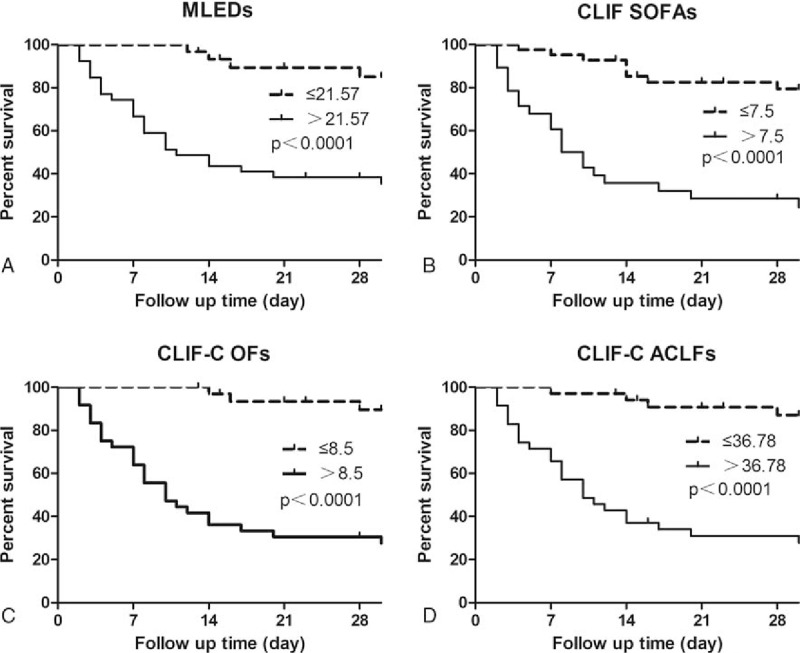
Kaplan–Meier survival curves by MELD (Standard), CLIF SOFA, CLIF OFs, and CLIF ACLF. ACLF = acute-on-chronic liver failure, CLIF SOFAs = CLIF sequential organ failure assessment score, MELDs = model for end-stage liver disease score.

The mortality rate within 28 days after hospital admission was 14% in no ACLF group, 20% in ACLF grade 1 group, 69% in ACLF grade 2 group, and 100% in ACLF grade 3 group, respectively. Overall, the 28-day mortality was 32% in all patients, and 100% in those with severe early course (ACLF-3). The AUROC of CLIF-C OFs (AUROC: 0.906, *P* = .0306, compared with MELDs) was higher than those of CLIF-SOFAs (AUROC: 0.876), CLIF-C ACLFs (AUROC: 0.858), and MELDs (AUROC: 0.838) for predicting short-term mortality (Fig. 2). The optimal cut-point for baseline MELDs in predicting death was 21.57, with 88.9% sensitivity and 67.2% specificity (Fig. 3A), and for CLIF-SOFAs 7.5 (74.1% sensitivity and 82.2% specificity, Fig. 3B), CLIF-C OFs 8.5 (92.6% sensitivity and 74.1% specificity, Fig. 3C) and CLIF-C ACLFs 36.78 (88.9% sensitivity and 72.4% specificity, Fig. 3D). The AUROC of CLIF-C OFs was 0.906 (95% CI: 0.8450–0.9679). Based on the optimal cut-off values, patients were further categorized into 2 groups, the group that CLIF-C OFs≥8.5, MELD≥21.57, CLIF-SOFA≥7.5, or CLIF-C ACLFs≥36.78, mean high mortality.

## 4 Discussion

The validity of the CLIF-C OFs model is upheld by this study, confirmed through independent analysis of an Asian CHB patients’ cohort with ACLF. In comparing CLIF-C OFs with other existing prognostic systems, its superiority in predicting short-term death within the 28-day of an acute episode is demonstrated. The fact that CLIF-C OF scores system reflects multiorgan functional declines (hepatic, renal, cerebral, coagulatory, circulatory, and respiratory), whereas models such as MELD, though widely used in clinical practice, reflect less organ function than CLIF-C OFs. Calculating CLIF-C OFs (range 6–18) is more convenient than CLIF-SOFAs, CLIF-C ACLFs, and MELDs. Our implementation of CLIF-C OFs within the critical 28 days of hospitalization was quite satisfactory. The validity of the CLIF-C OFs model is upheld by this study, confirmed through independent analysis of an Asian CHB patients’ cohort with ACLF. CLIF-C OFs is more accurately than other existing prognostic systems in predicting 28-day mortality in our study. Moreover, calculating CLIF-C OFs (range 6–18) is more convenient than CLIF-SOFAs, CLIF-C ACLFs, and MELDs. Our implementation of CLIF-C OFs within the 28 days for assessing outcome was quite satisfactory.

A previous study found that the following variables age, prothrombin activity, serum sodium, total bilirubin, hepatitis B e antigen positivity rate, and hemoglobin were significantly related to the prognosis of acute-on-chronic hepatitis B liver failure.^[13]^ Our study showed that the variables age, total bilirubin, creatinine, albumin, serum sodium, and INR were significantly related to the prognosis of CHB patients with ACLF, and high INR and lower albumin remained as independent factors associated with liver-related mortality. We assessed some novel models, CLIF-C OFs, CLIF-SOFAs, and CLIF-C ACLFs, for predicting the short-term mortality of ACLF in CHB patients, which was built at 2014 and had not been used in this field compared with the previous studies.

A previous study found that CLIF-SOFA enables more accurate prediction of short-term mortality in patients with acutely decompensated alcoholic cirrhosis than MELDs and CTP.^[14]^ Another study showed that the CLIF-C ACLFs may be more useful for predicting 28-day and 90-day mortality in ACLF cases than CTP, MELD, and MELD-sodium scores in alcohol-related ACLF.^[15]^ Our results in CHB patient with ACLF were similar to that in those previous studies, but the best performance was CLIF-OFs, not CLIF-SOFA or CLIF-C ACLFs.

We determined the cut-points of CLIF-C OFs. CLIF-C OFs ≥ 8.5 was at high risk of death, thus prioritizing patients for organ allocation. At scores of 8 to 10 (ACLF-2), aggressive management still might be in order, including early use of renal replacement treatment, extracorporeal liver support, measures to prevent hepatic encephalopathy, and broad-spectrum antibiotic prophylaxis. Our study confirmed results of the CANONIC study, which indicated that a negative history of prior acute decompensation is associated with higher mortality rates in patients with ACLF.^[11]^

Our study has some limitations. First, there may be bias in a single-center study. The CANONIC study found that the CLIF-C ACLFs at ACLF diagnosis is superior to the MELDs and MELD-Nas in predicting mortality.^[10]^ However, our study showed that the CLIF-C ACLFs was similar accurate in predicting short-term mortality compared with CLIF-SOFAs and CLIF-C OFs, due to the small sample size of this study. We used once-only scores in predicting short-term mortality, whereas serial delta scores might have been more useful in predicting the outcome in critically ill patients like ACLF. Second, we do not compare with other prognostic scoring systems, such as CTP and acute physiology and chronic health evaluation (APACHE II score). A recent study in which patients were defined as ACLF using the APASL criteria except for the inclusion of nonhepatic insults as acute events found that APACHE II score performed better than SOFA, CTP, and MELD.^[16]^ Moreover, it is anticipated that further critique and validation of emerging and relevant biomarkers will facilitate a composite score that, either alone or in combination with existing scoring systems such as CLIF-C ACLFs, will enable improved prognostication and targeting of therapy in ACLF.^[17]^ Third, genotypes of HBV are associated with disease progression and treatment responses. However, viral genotypes have diverse geographical distribution, and the genotypes of HBV are almost genotype B or C in our hospital according to a previous study, although we did not detect it in this study. Last, the cut-points generated from this study should be validated by others. Still, some study has demonstrated that assessment of ACLF patients at 3 to 7 days of the syndrome provides a tool to define the emergency of LT and a rational basis for intensive care discontinuation owing to futility.^[12]^ It may be useful for predicting the patients with ACLF for liver transplantation via accurately scoring system, which ultimately may give them better survival.^[18]^ These cut-points may help identify patients at high risk of early mortality, prompting more aggressive management.

In conclusion, CLIF-C OFs scoring of multiorgan failures best predicted short-term mortality in CHB patients with ACLF, compared with 4 other prognostic scoring systems. The 28-day mortality was accurately predicted via a convenient scoring system, CLIF-C OFs. Nonetheless, a prognostic model befitting Asian CHB patients with ACLF would be optimal, given issues of racial disparity, genomic difference, and cultural diversity.
